# Advances in the Synthesis of Heterocyclic Compounds and Their Applications

**DOI:** 10.3390/molecules30183723

**Published:** 2025-09-12

**Authors:** Ionel I. Mangalagiu, Mircea Darabantu

**Affiliations:** 1 Faculty of Chemistry, Alexandru Ioan Cuza University of Iasi, 11 Carol 1st Bvd, 700506 Iasi, Romania; 2 Department of Chemistry, Faculty of Chemistry and Chemical Engineering, University Babes-Bolyai Cluj-Napoca, 11 Aranyi Janos str., 400028 Cluj-Napoca, Romania

The synthesis of heterocyclic compounds remains a major goal in organic synthesis, especially because of their numerous applications in medicine, pharmacy, opto-electronics, agriculture, and related fields [1,2,3,4,5]. Subject to all evolutionary aspects of heterocyclic chemistry, modern approaches to heterocycles (reagents, methodologies, strategies, reaction mechanisms, chemical and instrumental auxiliaries, catalysts, etc.) nevertheless obey two crucial aspects, “selectivity” and “specificity” (including typical prefixes such as chemo-, regio-, or stereo-), as compulsory criteria [6,7,8,9,10,11]. Targeting heteromolecules and their applications in human life and in the environment, this Special Issue (S.I.) provides a current overview of the latest progress in synthetic methodologies and chemical technologies by updating relevant and important original contributions and reviews.

Contribution 1 is related to the synthesis and bioactivity of new boronic derivatives. Their antimicrobial activities (against *M. tuberculosis* and the fungal strains *C. albicans*, *T. mentagrophytes*, and *T. rubrum*) and anticancer properties (against oral squamous cell carcinoma cell lines) were determined, and some of the compounds were found to possess promising activities.

Contribution 2 focuses on the synthesis and spectral characterization of 3,5-bis-aminated pyrazolo[1, 5-*a*]pyrimidines using an innovative catalyzed Ullmann coupling methodology (CuI, microwave heating). Its advantages include very good yields, short reaction times, no toxic reagents, and a broad substrate scope.

Contribution 3 highlights the synthesis and spectral characterization of new tetracyclic heterosteroidal compounds, namely 14-aza-12-oxasteroids. The reaction pathway involves the Bucherer conversion of 2-naphthols to 2-naphthylamines, their subsequent regioselective C-acetylation (via the Sugasawa reaction), and borohydride reduction of the acetyl groups. Key intermediates of the resulting naphthalene amino-alcohols undergo double dehydration and subsequent double intramolecular cyclization in reaction with oxo-acids to afford the desired 14-aza-12-oxasteroids.

The electrochemical synthesis of 2-aminoxazole-based polycyclic compounds is another novel finding presented in the SI. Contribution 4 describes the electrochemical synthesis of *N*-arylnaphtho- and *N*-arylanthra[2, 3-*d*]oxazol-2-amines by reaction of 3-amino-2-naphthol or 3-amino-2-anthracenol with isothiocyanates in the presence of potassium iodide, using a graphite electrode as an anode and a platinum electrode as a cathode. The resultant naphthalene/anthracene-fused tricyclic and tetracyclic oxazoles exhibit extended π-conjugated skeletons and fluoresce in the 340–430 nm region.

Contribution 5 discusses the synthesis and bioactivity of some hybrid indole and 8-hydroxyquinoline derivatives linked to a di- or triaryl methane moiety. A direct synthetic protocol was also the most effective, and the isolated hybrid compounds were proven to have significant anticancer activity against the resistant colon adenocarcinoma cell line Colo320 and non-tumor fibroblast cells.

This SI has also welcomed theoretical research papers, such as Contribution 6, centered on calculating the premise of the regioselective synthesis of 3-nitro-substituted 2-isoxazolines via a molecular cycloaddition mechanism of the [3 + 2] type, involving nitro-substituted formonitrile *N*-oxide and electron-rich alkenes.

In Contribution 7, the authors report the synthesis and utility of some new 3,7-diheteroaryl-substituted 10-(3-(trimethylammonium)propyl)-10H-phenothiazine derivatives. They and their precursors exhibited reversible redox behavior with tunable potentials and blue to green-blue emissive aptitude. Some of the compounds demonstrated good antimicrobial activity against strains of *M. tuberculosis*, *A. baumannii*, *E. coli*, *S. aureus*, and *K. pneumoniae*.

The synthesis and bioactivity of some new 6-iodo-substituted carboxy-quinolines is discussed in Contribution 8. The study describes their one-pot, three-component reaction catalyzed by trifluoroacetic acid, which was efficient thanks to cost-effective catalysts, rapid response times, expeditious purification procedures, and high product yields. Their antimicrobial activity against strains of *K. pneumonie*, *S. epidermidis*, and *C. parapsilosis* was tested, with very promising results.

Contribution 9 concerns the synthesis and spectral characterization of new fused isoxazolidine/isoquinolinone and isoxazole/isoquinolinone hybrids. The reported strategy was facile and efficient, with general applicability, consisting of a three-step reaction sequence: a 1,3-dipolar cycloaddition, a Schmidt reaction, and an Ullmann-type cyclization.

Contribution 10 describes the synthesis and spectral characterization of a new library of C^3^-difluoromethyl carbinol-containing imidazo[1, 2-*a*]pyridines. The HFIP-assisted Friedel–Crafts reaction of difluoroacetaldehyde ethyl hemiacetal and imidazo[1, 2-a]pyridines revealed facile and efficient access to the desired compounds. The green synthetic protocol had a wide substrate scope and had several advantages: it was highly efficient, was carried out at room temperature, and involved no transition metals or oxidants.

Finally, the SI also includes two extensive reviews related to the synthesis of heterocyclic compounds and their applications.

Contribution 11 presents advances in the synthetic knowledge of various pathways leading to quinoline-4-ones, together with an overview of their structures, evolutionary development, and structure–activity relationships. Contribution 12 offers an interesting perspective with respect to some classes of quinoline derivatives and related structures (including 4-aminoquinolines, quinoline-4-ols, and fused and spiro-quinolines). The selected examples are mostly discussed in terms of 2-azidobenzaldehyde-based [4 + 2] annulations. In addition, evidence supporting the aptitude of 2-azidobenzaldehyde-initiated synthesis for one-pot stepwise synthesis or multicomponent reactions is presented. Some of the synthetic approaches included in this Special Issue provide novel pathways for quinoline synthesis and could also be used to obtain other heterocyclic compounds.
